# Reassessing the existence of soft X-ray correlated plasmons

**DOI:** 10.1038/s41467-023-39324-5

**Published:** 2023-10-24

**Authors:** Mohsen Moazzami Gudarzi, Seyed Hamed Aboutalebi

**Affiliations:** 1 National Graphene Institute, University of Manchester, Manchester, UK; 2 Department of Materials, School of Natural Sciences, The University of Manchester, Manchester, UK; 3 Condensed Matter National Laboratory, Institute for Research in Fundamental Sciences, Tehran, 19395-5531 Iran

**Keywords:** Optical physics, Materials for optics, Nanophotonics and plasmonics, Electronic properties and materials

**arising from** T. J. Whitcher et al. *Nature Communications* (2021) 10.1038/s41467-021-27182-y

Research on the complex dielectric functions of transition metal dichalcogenides (TMDs) including MoS2 has spanned half a century, employing both experimental and theoretical methods^1–7^, with the integrity of findings ensured through using optical sum-rules and the constraints of inertia and causality, despite occasional discrepancies in reported data^8,9^. Whitcher et al.’s study^10^ contributes to this body of knowledge by reporting the observation of anomalous soft X-ray correlated plasmons with low loss. Contrary to their findings, our analysis suggests that the data are inconclusive due to non-compliance with optical sum-rules and disagreement with expected optical constants for MoS2 based on tabulated atomic scattering factors of Mo and S^11^. These discrepancies signal the necessity for a critical reassessment of the evidence for soft X-ray correlated plasmons in TMDs.

We would like to cite measurements of optical constants of bulk MoS_2_ acquired using various techniques such as reflectivity^6^, electron energy-loss spectroscopy^2,3^ and inelastic X-ray scattering^7^ over the course of last 50 years. In all these measurements, at room temperature, two distinctive plasmonic peaks at 8.7–8.9 and 23–23.5 eV have been consistently observed. These observations agree with the dispersion theory of plasmon excitation for semiconductors^2,3^. However, the room temperature loss function of MoS_2_ obtained by ref. ^10^ differs significantly from these measurements. Moreover, they report an anomalous shift of plasmonic peaks at lower temperatures of 150 and 100 K which is not consistent with the loss functions reported for layered TMDs over the same temperature range^1,12^. (Please see panel (d) of Figures 7 to 10 in ref. ^1^ and panel (d) of Figures 3 and 4 in ref. ^12^). Given that the plasmonic peaks in semiconductors are controlled by the valence electron density (which is practically temperature-independent), this shift is unexpected.

Moreover, the optical constants of materials, in general, are limited by causality which mandates these constants follow a set of sum rules, among them the *f*-sum rule, which states:^9^

1 $${\int }_{0}^{{E}_{{cut}-{off}}}\omega \cdot {\varepsilon }_{2}\left(\omega \right)d\omega=-{{\varepsilon }_{b}}^{2}{\int }_{0}^{{E}_{{cut}-{off}}}\omega \cdot {{{{{\rm{Im}}}}}}\left({\varepsilon }^{-1}\left(\omega \right)\right)d\omega=\frac{\pi }{2}{{\omega }_{p}}^{2}$$

where *ε*_2_ and Im(*ε*^−1^) are imaginary part of the dielectric function and loss function, respectively. *ω*_*p*_ is the plasma frequency and ${\omega }_{p}=\sqrt{\frac{n{e}^{2}}{{m}_{e}{\varepsilon }_{0}}}$, also given by eq. (5) of ref. ^10^. *ε*_*b*_ is the background dielectric constant induced by absorption bands above the cut-off energy, *E*_cut-off_, which is close to unity for large cut-off energies, like soft X-ray. Therefore, the effective number of electrons computed from *ε*_2_ is always larger than those obtained from loss function, and at high enough photon energy both these values converge^9^. Our analysis of the data reported by the authors shows that the dielectric function at 77 K, for instance, leads to *ε*_*b*_ of 0.92 at 45 eV (Fig. 1a). It should be noted that by definition the dielectric constant of vacuum at any frequency is unity. This implies *ε*_2_ should be negative at certain region for *ε*_*b*_ to be below unity, meaning that light not only does not dissipate but intensifies. This is against the energy conservation and is physically impossible. At room temperature, *ε*_*b*_ is found to be 1.25 at 45 eV, which is much larger than the expected value of 1.043 (See Supplementary Note 1 and Supplementary Fig. 1 for details). Interestingly, our analysis of the published data demonstrated an unphysically large number of valence electrons. At room temperature and by integrating *ε*_2_ component, the effective number of electrons is nearly 42, meaning that there are only 32 more electrons left to contribute to the bands above 45 eV (total number of electrons in MoS_2_ is 74 in non-relativistic limit) (Fig. 1a). This value (42) is far above the nominal number of valence electrons for MoS_2_ according to the classical atomic orbital theory (18 electrons, see electron configurations of Mo and S in inset of Fig. 1c).

**Fig. 1:**
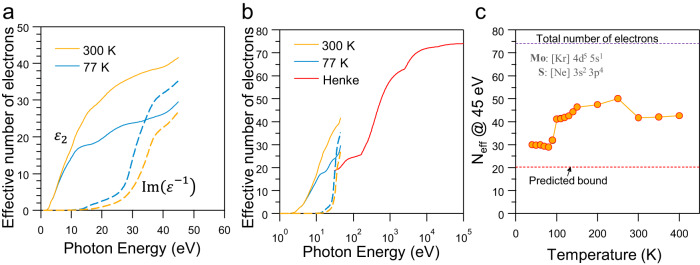
Mismatch of the number of electrons in MoS2 with nominal values. **a** This shows the evolution of the effective number of electrons (*N*_eff_) contributing to the absorption bands for two sets of data at 300 and 77 K. *N*_eff_ are computed from both *ε*_2_ (solid lines) and Im(*ε*^−1^) (dashed lines). **b** This shows the same data along with the *N*_eff_ profile expected for MoS_2_ with density of 5.0 g/cm^3^ (red line), using the tabulated atomic scattering factors for Mo and S^11^. The high energy tail was set to 74, the nominal number of electrons for MoS_2_. **c** This shows the *N*_eff_ at 45 eV (orange circles) based on the reported integrated optical conductivity by ref. ^10^. The predicted N_eff_ bound at this energy is 20.2.

In principle, the classical theory slightly underestimates the number of valence electrons and one expects marginally larger values in real materials as dictated by Pauli exclusion principle^13^. One can count more precisely the expected number of electrons up to this photon energy (45 eV) using the atomic scattering factors for Mo and S and the density of 2H-MoS_2_^8,11^. We found that the correct effective number of electrons to be about 20.2 at 45 eV, which is less than half of the number of electrons obtained from the authors’ data at room temperature (Fig. 1b). This deviation is even more significant for the temperature range of 150–250 K based on the authors’ analysis (Fig. 3b of their paper and Fig. 1c). We must stress that in the above soft X-ray photon energy range, condensed materials in general show ‘atomic-like’ behaviour, where they can be modelled as non-interacting atoms, except close to absorption edges^11^. This additive behaviour has been experimentally confirmed in various materials and it is valid at high photon energies, typically above plasma frequency where all bands originated from valence electrons are exhausted^14^.

In addition, above 45 eV, it is mostly the core-electrons that contribute to the polarisation, and given the very high excitation energy of these electrons, it is not possible for temperature variation, at least in the argued range, i.e. 3.5–35 meV, to cause any changes to the effective number of core electrons. This combined with that fact that the change in the mass density of 2H-MoS_2_ is below 1% in the specified temperature range (See Supplementary Note 2 and Supplementary Fig. 2), implies electron density stays rather constant within this temperature range. Therefore, the reported data do not match the possible optical constants for 2H-MoS_2_ and contradict the fundamental limits imposed by causality^9^. Also, despite claiming consistency with Kramers–Kronig relations, such as:

2 $${\varepsilon }_{1}\left(0\right)=1+\frac{2}{\pi }{\int }_{0}^{{{\infty }}}\frac{{\varepsilon }_{2}(\omega )}{\omega }d\omega,$$

we found, for instance, the electronic dielectric constant, *ε*_1_(0), at 300 K to be about 23.3 using the above relation^8^, which is nearly four units larger than the calculated values by authors (Fig. 1b of ref. ^10^) and 7 units larger than the previous reports^8,15^.

Finally, we would like to call attention to the abnormally high reflectivity reported by the authors at energies between 25 and 35 eV, especially at high temperatures, ranging between 40 and 20%. This is nearly one order of magnitude larger than previous reports for bulk MoS_2_^4^ and any other layered TMDs^5,8^ that we are aware of. Once again, this inconsistency with previous reports is not inherently forbidden. However, based on the normalisation protocol reported by the authors themselves, using the tabulated values for Mo and S^11^, and the mass density of 2H-MoS_2_ (Fig. 2a), we found the reflectivity to be significantly smaller (exceeding 14 times lower) than the reported values by ref. ^10^ at all photon energies between 30 and 45 eV (Fig. 2b). Therefore, the validity of the data is questionable.

**Fig. 2:**
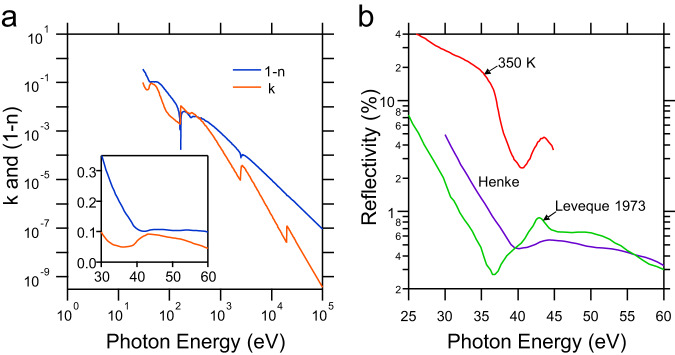
Abnormally high reflectivity of MoS2 in vacuum UV region. **a** This shows optical constants of MoS_2_ with density of 5.0 g cm^−3^, calculated from the atomic scattering factors of Mo and S^11^. Inset shows the data in the region relevant to the measurements reported in ref. ^10^. **b** This compares the reflectivity coefficient reported in ref. ^10^ at 350 K, and those obtained from constants shown in (**a**), named as Henke. We also included the experimental data by ref. ^4^ on reflectivity of MoS_2_, which show an agreement (at higher energies) with those reflectivity data obtained from atomic scattering factors, but significantly smaller than those in ref. ^10^.

Our analysis and arguments outlined above strongly suggest that the anomalous correlated plasmons reported by the authors are artificial and most likely are a result of erroneous normalisation of the data. We note that the validation of the consistency of the optical constants of materials, especially those obtained by compounding spectra, against the physical constraints is of paramount importance and a matter that should not be taken lightly. We showed that these procedures to evaluate self-consistency, despite being developed decades ago, has not been checked. This calls for revisiting the original measurements and claims regarding the anomalous correlated plasmons.

### Supplementary information

Supplementary Information

## Data Availability

All data generated or analysed during this study are included in the published article. All relevant processed data are available from the authors upon reasonable request.
