# Supplementary material for: Reassessing the existence of soft X-ray correlated plasmons
Source: Nat Commun. 2023 Oct 24;14:6753. doi: 10.1038/s41467-023-39324-5 (PMC10598222; doi:10.1038/s41467-023-39324-5)
Supplement: Supplementary file 1 — Supplementary Information [file 41467_2023_39324_MOESM1_ESM.pdf]

Supplementary information

Reassessing the Existence of Soft X-Ray Correlated Plasmons

Mohsen Moazzami Gudarzi[1,2*], Seyed Hamed Aboutalebi[3]

[1] National Graphene Institute; University of Manchester, Manchester, UK.

[2] Department of Materials, School of Natural Sciences; The University of Manchester, Manchester, UK.

[3] Condensed Matter National Laboratory; Institute for Research in Fundamental Sciences, Tehran, 19395-5531, Iran.

Supplementary Note 1:

- **Computation of background dielectric constant:**

We first calculated the imaginary part of the dielectric function using the atomic scattering factors of Mo and S [1], and the density of 2H-MoS_2. The optical constants are given in Figure 2a of the main text ($\varepsilon_2 = 2nk$). Then using the Kramers-Kronig relation, the background dielectric constant is given as[2]:

$$\varepsilon_b(\xi) = 1 + \frac{2}{\pi}\int_{\xi}^{\infty} \frac{\varepsilon_2(\omega)}{\omega} d\omega \quad (1)$$

Supplementary Figure 1. shows the variation of ε_b as a function of photon energy where it converges to unity at high photon energies. ε_b at 45 eV is 1.043.

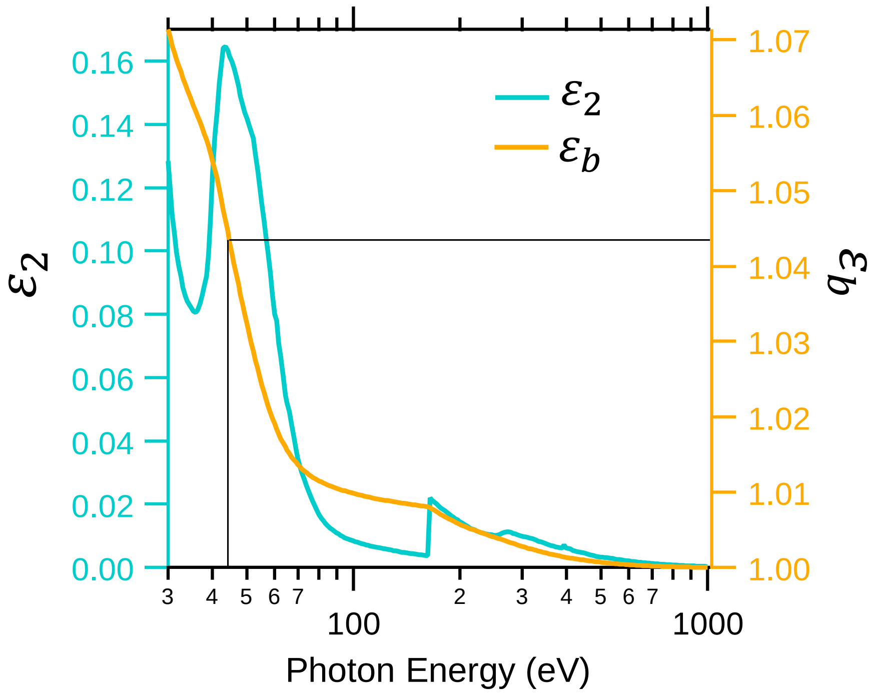

Supplementary Figure 1. Imaginary part of the dielectric function (ε_2) and background dielectric constant (ε_b) of 2H-MoS2.

Supplementary Note 2:

- **Density of 2H-MoS_2 versus temperature:**

As the plasma frequency depends on the electron density, we have calculated the density of the 2H-MoS_2 crystals using the available cell parameters as a function of temperature. Data on cell parameters are reported in refs.[3,4] We then converted these data to density using the given crystal structure (Supplementary Figure 2). The computed densities versus temperature were fitted to a polynomial function and used to calculate the effective number of electrons from the plasma frequencies.

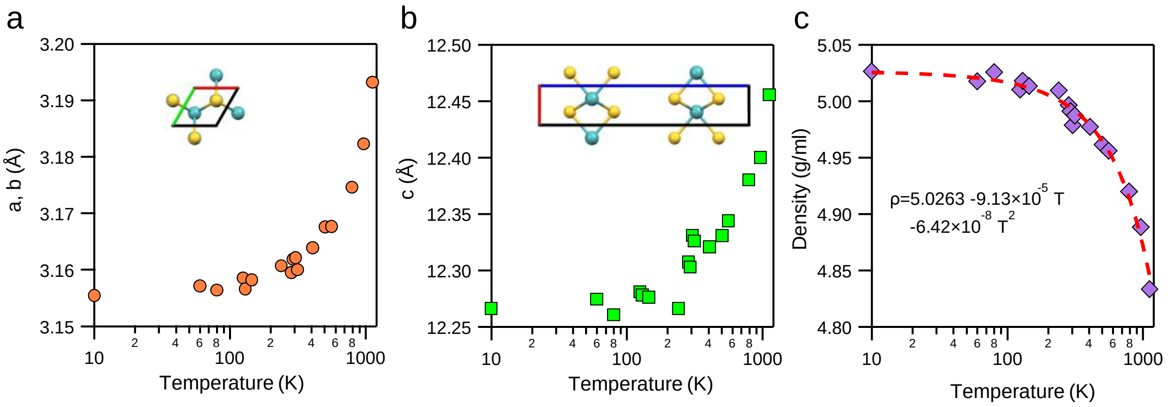

Supplementary Figure 2. Density of 2H-MoS_2 as a function of temperature. Panels (a) and (b) show the cell parameters for crystal structure of 2H-MoS_2 which are obtained from X-ray powder diffraction measurements.[3,4] Panel (c) shows the computed densities for various temperatures and the polynomial fit to the data. Density close to room temperature is found to be 4.99 g/ml. The average density of 2H-MoS_2 at room temperature is 5.00 ± 0.031 g/ml (N=26) based on analysis of data compiled in SpringerMaterials database.[5]

References:

1 Henke, B. L., Gullikson, E. M. & Davis, J. C. X-ray interactions: photoabsorption, scattering, transmission, and reflection at E= 50-30,000 eV, Z= 1-92. *At. Data Nucl. Data Tables* **54**, 181-342 (1993).

2 Smith, D. Y. in *Handbook of Optical Constants of Solids* (ed Edward D. Palik) 35-68 (Academic Press, 1985).

3 Murray, R. & Evans, B. The thermal expansion of 2H-MoS2 and 2H-WSe2 between 10 and 320 K. *J. Appl. Crystallogr.* **12**, 312-315, doi:https://doi.org/10.1107/S0021889879012528 (1979).

4 El-Mahalawy, S. H. & Evans, B. L. The thermal expansion of 2H-MoS2, 2H-MoSe2 and 2H-WSe2 between 20 and 800°C. *J. Appl. Crystallogr.* **9**, 403-406, doi:https://doi.org/10.1107/S0021889876011709 (1976).

5 MoS2 Crystal Structure: Datasheet from "PAULING FILE Multinaries Edition – 2012" in SpringerMaterials (Springer-Verlag Berlin Heidelberg & Material Phases Data System (MPDS), Switzerland & National Institute for Materials Science (NIMS), Japan).
